# White Matter Injury in Preterm Infants: Pathogenesis and Potential Therapy From the Aspect of the Gut–Brain Axis

**DOI:** 10.3389/fnins.2022.849372

**Published:** 2022-04-29

**Authors:** Yu He, Yuni Zhang, Fang Li, Yuan Shi

**Affiliations:** ^1^Department of Neonatology, Children’s Hospital of Chongqing Medical University, Chongqing, China; ^2^Chongqing Key Laboratory of Pediatrics, Children’s Hospital of Chongqing Medical University, Chongqing, China; ^3^Ministry of Education Key Laboratory of Child Development and Disorders, National Clinical Research Center for Child Health and Disorders, China International Science and Technology Cooperation Base of Child Development and Critical Disorders, Chongqing, China

**Keywords:** white matter injury, oligodendrocyte precursor cells, hypomyelination, microglia, inflammation, short-chain fatty acids

## Abstract

Very preterm infants who survive are at high risk of white matter injury (WMI). With a greater understanding of the pathogenesis of WMI, the gut microbiota has recently drawn increasing attention in this field. This review tries to clarify the possible mechanisms behind the communication of the gut bacteria and the immature brain *via* the gut–brain axis. The gut microbiota releases signals, such as microbial metabolites. These metabolites regulate inflammatory and immune responses characterized by microglial activation, which ultimately impact the differentiation of pre-myelinating oligodendrocytes (pre-OLs) and lead to WMI. Moreover, probiotics and prebiotics emerge as a promising therapy to improve the neurodevelopmental outcome. However, future studies are required to clarify the function of these above products and the optimal time for their administration within a larger population. Based on the existing evidence, it is still too early to recommend probiotics and prebiotics as effective treatments for WMI.

## Introduction

The global incidence of preterm births has been increasing and has accounted for 11% of all live births worldwide in 2010 (Blencowe et al., 2013). Although the survival rate of preterm infants has notably increased due to medical advancements, unfortunately, many survivors, particularly very preterm infants (less than 28 gestational weeks), are present with impaired neurodevelopment later in life, which may lead to subsequent economic burden. White matter injury (WMI) is a unique and common form of brain injury in preterm infants, especially in those born before 28 gestational weeks (Rantakari et al., 2021). Accumulating evidence over recent decades indicates that the gut microbiota has a critical role in WMI as it regulates oligodendrocyte (OL) maturation and inflammation. Herein, we discussed the crosstalk of preterm WMI and the gut microbiome and elucidated the potential mechanisms by which the gut bacteria and the immature brain communicate with each other. This communication occurs *via* several pathways involving microbial metabolites and inflammatory and immune responses characterized by microglial activation, which ultimately impact the differentiation of pre-myelinating OLs (pre-OLs) and lead to WMI. Currently, the treatment of WMI is limited to preventive management rather than targeted therapies. In this review, we summarized the updated evidence of microbial-based intervention and treatment (e.g., probiotics and prebiotics) for preterm WMI and tried to analyze the neuroprotective efficacy of probiotics and prebiotics in very preterm infants.

## Overview of White Matter Injury

At present, brain injuries after preterm birth involve not only white matter (Pierrat et al., 2017) but also gray matter (Ophelders et al., 2020), even cerebellum (Van Kooij et al., 2012). In this review, we discussed WMI, which is a unique form of brain injury in very preterm infants and causes neurological complications, such as cerebral palsy, audiovisual dysfunction, and cognitive impairment. The most serious outcome is periventricular leukomalacia, which appears as large areas of cystic necrosis adjacent to the ventricular wall. The patterns of WMI mainly comprise two major forms of pathology, namely, necrotic and diffuse WMI (Back, 2015, 2017). Necrotic WMI can be divided into two types, namely, cystic and non-cystic or microscopic WMI, which has a diameter of less than 1 mm. Although cystic WMI was the more common form of WMI in the last few decades, its current prevalence has markedly declined to <5% due to improvements in perinatal care (Groenendaal et al., 2010). Diffuse WMI, however, is reportedly the most common form of preterm brain injury in recent cohorts of preterm newborns (Back and Miller, 2014; Back, 2015). As reviewed by Back (2017), diffuse WMI is featured by selective death of pre-OLs in contrast to necrotic WMI. Multiple perinatal insults selectively trigger pre-OL injury, whereas compensatory pre-OLs regenerated in diffuse WMI fail to differentiate into mature OLs (Ophelders et al., 2020). Hence, impaired myelination in diffuse WMI is caused by arrested pre-OL maturation rather than white matter necrosis and displays in MRI with decreased white matter volume, thinning of the white matter tracts, and diffuse microscopic injury (Rutherford et al., 2010; Alexandrou et al., 2014).

Although the pathogenesis of WMI remains speculative, there are several perinatal triggers associated with WMI in very preterm infants, such as antepartum infection (Elovitz et al., 2011; Chau et al., 2014; Jenster et al., 2014; Yap and Perlman, 2020) (i.e., maternal chorioamnionitis), hypoxic–ischemic injury (Jellema et al., 2013; Wang et al., 2019), multiple postnatal detrimental exposures (Wyatt, 2007; Albertine, 2012; Ophelders et al., 2020) (i.e., mechanical ventilation, hemodynamic instability, oxidative stress, and neonatal sepsis), and early nutritional intake (Beauport et al., 2017; Schneider et al., 2018; Brandt et al., 2021; Hortensius et al., 2021). Moreover, the combination of immature cerebrovascular anatomy, disturbed autoregulation, and extrinsic insult exposure, such as hypoxia, ischemia, and inflammation, predisposes the vulnerable underdeveloped brain to impairment (Schneider and Miller, 2019). A period of persistent placental insufficiency and moderate hypoxemia during the last one-third of the gestation period hampers the brain development of a sheep fetus, particularly, the myelination of cortical white matter (Mallard et al., 1998; Duncan et al., 2004). Notably, WMI in very preterm infants is different from hypoxic–ischemic encephalopathy in full-term infants.

A remarkable feature of preterm WMI is hypomyelination. Normal myelination is critical for axonal conductivity and further plays an essential role in cerebral cortical development (Volpe, 2019) and learning and memory later in life (Wang et al., 2020). The pre-OLs are particularly vulnerable to insults like hypoxia and inflammation after preterm birth and are the major cellular targets in the preterm brain (Volpe et al., 2011; van Tilborg et al., 2018). Immature pre-OLs, migrating from OL precursor cells (OPCs), finally turn into mature OLs that produce the myelin used for encapsulating axons (van Tilborg et al., 2018). In preterm WMI, OPCs move into the regions of white matter, where they differentiate into pre-OLs as a supplement to OLs. However, these newly generated pre-OLs fail to differentiate into mature myelinating OLs (Segovia et al., 2008). This leads to a reduction in OLs in WMI, which eventually leads to hypomyelination.

Regarding the mechanisms that lead to the maturation arrest or death of OLs, oxidative stress is among the major causes. During the perinatal period, the excessive free radicals released after inflammation, hypoxia, and ischemia–reperfusion may disrupt the regular redox state of cells, thus increasing oxidative stress and its associated toxic effects. Pre-OLs are susceptible to oxidative stress (Sheldon et al., 2004; Back et al., 2005). Genes that regulate the maturation of OLs are activated by oxidative stress. Oxidative stress can hinder the differentiation of OLs by promoting global histone acetylation (French et al., 2009; Dewald et al., 2011). Oxidative stress can also cause the death of pre-OLs by activating the caspase systems (Back et al., 2005).

Furthermore, inflammation has recently drawn increasing attention for its role in brain injury (Hagberg et al., 2015). Treating mice with interleukin-1β (IL-1β) led to an increase in the number of unmyelinated axons and caused WMI. We assumed that this is because IL-1β inhibits the maturation of OLs by disrupting several transcription factors that could regulate the maturation of OLs (Favrais et al., 2011). In addition, tumor necrosis factor (TNF) is reportedly a key mediator in promoting lipopolysaccharide-induced death of OLs (Kim et al., 2011).

Moreover, the arrested maturation of pre-OLs is accompanied by microglial activation. The cerebral white matter is predominantly populated with microglia at 20–35 weeks’ gestation (Billiards et al., 2006), a period when numerous critical developmental events are occurring and when various perinatal insults (e.g., hypoxia, ischemia, and inflammation) are presented to trigger WMI. Microglia can be activated by recognizing various signals (primarily PAMPs and DAMPs) produced by cell death and inflammation (Ziemka-Nalecz et al., 2017). The microglia could be divided into active pro-inflammatory state (M1) and anti-inflammatory state microglia (M2) (Hammond et al., 2018). The activated M1 microglia act like macrophages, which can phagocytize, proliferate, and migrate into the injured areas (Bokobza et al., 2019). These cells can produce free radicals and generate pro-inflammatory cytokines, which can further exacerbate neuroinflammation and cause insults to OLs and neurons. Conversely, the M2 microglia can supply iron and increase the expression of PDGF-AA, VEGF, and IGF-1 to maintain OPC survival and promote OPC maturation (Todorich et al., 2009; Pang et al., 2013). Thus, diversion of microglia not in an active pro-inflammatory state (M2) to a phenotype with pro-inflammatory (M1) function could lead to the disturbed maturation of OLs observed in WMI. In addition to OL and axonal development, microglia take part in several crucial developmental events, such as synaptic formation, pruning, and plasticity (Hammond et al., 2018).

In addition to the innate immune system, the adaptive immune system also participates in WMI. The activation of T helper cells is demonstrated to be toxic to OLs (Horiuchi et al., 2006). Other studies have also revealed that T cells may influence the myelination in WMI in very preterm neonates (Horiuchi et al., 2006; Albertsson et al., 2014; Ortega et al., 2016).

These factors include pro-inflammatory cytokines, activated immune cells, and oxidative stress, which altogether lead to the maturation arrest of OLs and subsequently hypomyelination. Moreover, they shift the microglia into a pro-inflammatory state, which can further cause damage to OLs and can result in WMI.

## Major Events in Brain Development Occurring in Sync With Changes in Intestinal Microbiome

**FIGURE 1:**
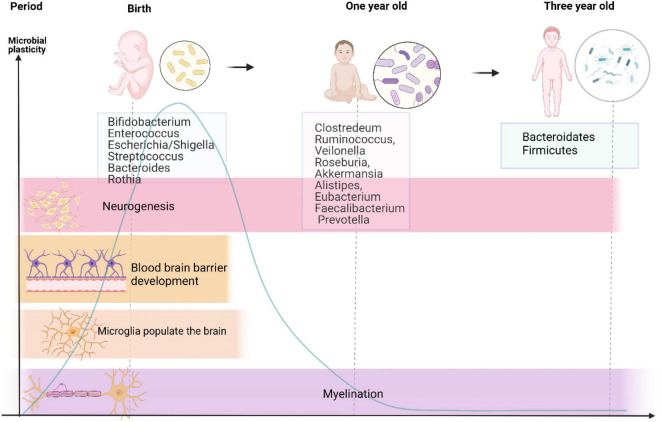
Developmental trajectories of gut microbiome and major neurodevelopmental events from birth to 3-year-old. This graph reveals changes in gut microbiota from birth to 3-year-old accompanied by major processes in neural development, suggesting that the developmental trajectory of the nervous system seems to overlap with that of the intestinal flora as life progresses. There exists a sensitive period in the gut–brain axis development, which is present with high microbial plasticity and critical for neurodevelopment. Microbial taxa in the middle are the predominant ones at different stages of life.

Neurodevelopment is a complicated and fluctuant process that is governed by multiple factors. Premature birth coincides with a crucial period when the brain undergoes several principal developmental events. In addition, these events were reported to be influenced by signals from the gut microbiota. There is no consensus on whether the fetus contains or is devoid of bacteria. Recent studies have reported the presence of microbiota in the placenta and amniotic fluid, indicating that the womb is not sterile (Perez-Muñoz et al., 2017; Bolte et al., 2022). However, others have questioned this evidence because of lacking controls of contamination (Theis et al., 2019) and have maintained that the fetus is living in a sterile environment. Anyway, early life exposures during pregnancy and lactation lead to alterations in microbiome throughout the life of offspring (Bolte et al., 2022). Because of individual variations, it is challenging to precisely define the composition of healthy microbiota during early life. However, as shown in Figure 1, it is well known that microbiota in neonates seem to follow certain developmental trajectories. Shortly after birth, the intestine is predominantly colonized by *Enterobacteriaceae*, *Clostridiaceae*, and *Bifidobacteriaceae*, and has low abundance of *Lachnospiraceae* and *Ruminococcaceae* (Bokulich et al., 2016; Yassour et al., 2016; Chu et al., 2017). Besides, Bäckhed et al. (2015) identified that the intestine of a neonate is aerobic and colonized with *Bifidobacterium*, *Enterococcus*, *Escherichia/Shigella*, *Streptococcus*, *Bacteroides*, and *Rothia*, and reflects the intestinal microbial composition of their mothers. As the neonate matures, bacteria proliferate dramatically and consume large amount of oxygen in the intestinal tract, forming an environment conducive to the colonization of anaerobic bacteria (Koenig et al., 2011). At the age of 1 year, kids are inhabited by *Clostridium*, *Ruminococcus*, *Veillonella*, *Roseburia*, *Akkermansia*, *Alistipes*, *Eubacterium*, *Faecalibacterium*, and *Prevotella* (Bäckhed et al., 2015). During the first year of life, the infants experience weaning, a shift to solid food intake, and rapid development of the brain. Strict anaerobes subsequently dominate the gut, and by around 3 years of age, an increased diversity of *Bacteroidetes* and *Firmicutes* similar to adulthood levels is noted (Koenig et al., 2011). Meanwhile, the infant microbiome during this period is susceptible to various perturbational factors, such as prenatal factors (e.g., maternal and intrauterine microbiome, diet, and stress), mode of delivery (Dominguez-Bello et al., 2010), gestational age (Fouhy et al., 2019), and some postnatal factors (Zhong et al., 2019) (antibiotics, breast feeding vs. formula feeding, and NICU environment). Recent research also found that basic neurodevelopmental processes coexist with the shift of intestinal microbiota (Lu et al., 2018). Research indicates that gut microbiota participates in perinatal brain development, including neurogenesis (Möhle et al., 2016), microglial maturation (Matcovitch-Natan et al., 2016), blood–brain barrier (BBB) development (Braniste et al., 2014), and myelination (Gacias et al., 2016), which persists into adulthood, as shown in Figure 1, and will be explained in the following paragraphs. Here, germ-free (GF) mice provide strong evidence for the role of gut microbiota in neural development.

Neurogenesis is influenced by the gut microbiota, demonstrated by the evidence that neurogenesis differs between GF mice and specific pathogen-free (SPF) mice. Neurogenesis in the dorsal hippocampus was increased in adult GF mice when compared with SPF mice. In addition, adult mice treated with antibiotics were found decreased hippocampal neurogenesis, which is mediated by Ly6Chi monocytes (Ogbonnaya et al., 2015; Möhle et al., 2016). GF mice also showed increased hippocampal and amygdala volumes (Ogbonnaya et al., 2015). Moreover, the brain-derived neurotrophic factor, which governs neuronal survival, was reportedly decreased in GF mice when compared with SPF mice (Ichim et al., 2012). These data provide evidence that intestinal microbes can influence the differentiation of neurons and ultimately brain development and health. Unlike neurons, microglia are responsible for immune defense in the central nervous system and play a key role in brain development, such as OL maturation and synaptic development mentioned earlier (Erny et al., 2015). Matcovitch-Natan et al. (2016) identified three stages of microglia maturation, which are characterized by transcriptionally gene expression and associated with chromatin changes, coinciding with the brain development. Furthermore, they found that the normal development of microglia is influenced by the gut microbiome. GF mice harbor microglia which exhibited dysregulation of a variety of genes associated with the mature adult phase and proper immune response (Matcovitch-Natan et al., 2016). These immature microglia have a weakened capacity to protect against infection and recognize microbially associated molecular patterns (MAMPs). However, this study is conducted in mice, while the picture is more complicated within the human body. Interestingly, such abnormal responses can be reversed by the supplementation of microbiota-derived short-chain fatty acids (SCFAs) (Erny et al., 2015).

The BBB starts to develop during the period of gestational life. However, BBB permeability in GF mice is dramatically increased compared to SPF mice and is accompanied by lower expression of the tight junction protein (i.e., occludin) (Braniste et al., 2014). Furthermore, it has been suggested that BBB permeability can be rescued by postnatal colonization of the microbiota (Braniste et al., 2014), indicating that microbiota can have a pivotal effect on the development of the BBB. Interestingly, the BBB works as an intermediate pathway for the gut–brain axis (Louveau et al., 2015).

As for myelination, OPCs differentiate into OLs and myelinated axons. This occurs in the frontal lobes of the cerebral cortex and is essential for learning and memory later in life. Similarly, myelination is also affected by the colonization of GF animals or antibiotic treatments. More details on the participation of the gut–brain axis in myelination are elucidated in the following section.

In summary, there are some sensitive periods in the development of gut–brain axis, which are critical for neurodevelopment. For example, recolonization with normal microbiota in GF mice restored the social deficits when performed at weaning but failed when performed 4 weeks later in adulthood (Buffington et al., 2016). However, even with recolonization as early as weaning (Chu et al., 2019), several functions in GF animals cannot be reversed, indicating that the gateway for microbial effect on these brain functions has already stopped by the time of weaning. In human studies, compared to antibiotic exposure at later stages in life, antibiotic exposure early in life has been reported to be detrimental to cognitive development (Slykerman et al., 2019). These results suggest that the gut–brain axis plays a crucial role in brain development.

## Potential Mechanisms of Gut–Brain Axis in Preterm White Matter Injury

### The Function of Gut Microbiota and Metabolites in Myelination

The differentiation of pre-OLs and myelination in WMI is crucial, and the gut microbiota may influence the myelination in different ways. Chen et al. (2019) reported that the use of antibiotics would induce demyelination, whereas, Hoban et al. (2016) reported that GF mice exhibited hypomyelination with upregulation of genes related to myelination and myelin plasticity. In addition, transplantation of conventional microbiota could reverse these changes in these myelination-activated genes (Hoban et al., 2016; Chen et al., 2019). These studies emphasize the importance of gut microbiota in the homeostasis of myelination. Microbiota-derived metabolites are the bridge of the microorganisms and the host since the changes of microbial metabolites reflect the variation of microbiota, environment, and host (Krautkramer et al., 2021). In spite of the complexity in the crosstalk of microbiota and host, knowledge of metabolites could provide a direct feature of the host–microbiota system as a whole, and the function of the microbiota had been explored in several neonatal diseases, such as necrotizing enterocolitis (NEC) (He et al., 2021; Renwick and Stewart, 2022) and bronchopulmonary dysplasia (Zhao et al., 2018). In the gut–brain axis, the metabolites released from microbiota also played an important role in myelination of prematurity. Gacias et al. (2016) revealed that microbiota could change gene expression related to myelination *via* the effect of their cresol metabolites on OLs (Gacias et al., 2016). This special metabolite may inhibit the differentiation of pre-OLs into mature OLs (Sauma and Casaccia, 2020). In addition, the treatment of SCFAs could suppress demyelination and enhance remyelination independently of microglia as depletion of microglia does not affect the enhancement of remyelination (Chen et al., 2019). SCFAs, including acetate, butyrate, and propionate, are the main products of fermented diet fibers and are the candidate metabolites involved in the gut–brain axis (Stilling et al., 2016). SCFAs are primarily produced by the bacteria that belong to Firmicutes and Bacteroidetes phyla, which are less abundant in very preterm babies (Arboleya et al., 2012; Zhang et al., 2015). One known potential mechanism for the SCFAs’ role in WMI is their ability to inhibit histone deacetylases (HDACs) (Waldecker et al., 2008; Soliman and Rosenberger, 2011). The expression of various genes can be regulated by acetylation of the histones, which would activate the transcription process. In turn, HDAC would lead to transcriptionally silenced chromatin. In the presence of SCFAs, transcriptionally silenced chromatin caused by HDAC would be reversed as SCFAs are natural HDAC inhibitors (Dalile et al., 2019). In a neonatal hypoxic–ischemic rat model, a marked decrease in the number of OPCs after hypoxic–ischemic insult could be reversed by treatment with sodium butyrate *via* the effect of sodium butyrate on acetylated histone 3 (Ziemka-Nalecz et al., 2016). This result is in agreement with the findings of another study which reported that treatment with sodium butyrate can induce histone acetylation and can further aid in the differentiation and maturation of OLs, consequently conferring protection against WMI in a neonatal hypoxic–ischemic rat model (Huang et al., 2018).

### The Inflammatory Regulation of Gut Microbiota in White Matter Injury

Since accumulating evidence suggests that inflammation is a key mediator in WMI in very preterm infants, regulating inflammation may be the bridge of microbiota and preterm brain injury.

**FIGURE 2:**
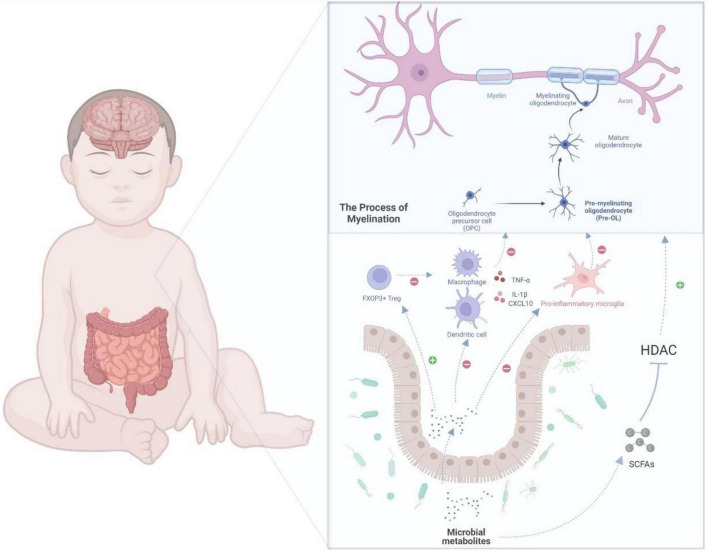
A potential gut–brain pathway through which the microbial metabolites might modulate preterm white matter injury. Myelination, which is determined by the normal maturation of pre-OLs, plays a key role in WMI. In the gut–brain axis, the microbial metabolites, such as SCFAs, which inhibit HDAC, could regulate oligodendrocyte maturation and subsequently myelination. Moreover, SCFAs can also regulate immune cells, such as macrophages, T cells, microglia, and DCs. For example, SCFAs could inhibit the differentiation of microglia into pro-inflammatory type, which may damage the maturation of pre-myelinating oligodendrocyte. In addition, SCFAs could weaken the production of cytokines, such as interleukin-1β and CXCL10. By modulating the immune cells and cytokines, microbial metabolites can further regulate oligodendrocyte maturation and protect extremely preterm infants from WMI. DCs, dendritic cells; IL-1β, interleukin-1; Pre-OLs, pre-myelinating oligodendrocytes; OLs, oligodendrocytes; WMI, white matter injury; HDAC, histone deacetylases.

An equilibrium relationship between the gut microbiota and immune system is critical to inhibit immoderate inflammation. However, the immune system and gut microbiome in very preterm infants are not fully developed, which may lead to excessive inflammation and negatively affect the brain. A recent study found that *Klebsiella* overgrowth with subsequent alterations, such as increased γδ T-cell levels, are highly predictive of preterm brain damage and are associated with a pro-inflammatory immunological environment in the central nervous system (Seki et al., 2021). In addition, McMurran et al. (2019) reported that gut microbiota could influence the inflammation in the central nervous system. Antibiotic-treated mice showed activated microglia and macrophages after demyelination (McMurran et al., 2019). As mentioned earlier, the microbe-derived products of fermented dietary fiber, SCFAs, can ameliorate microglial dysfunction in GF and antibiotic-treated animals (Erny et al., 2015), indicating that microbial metabolites may be a common mechanism for gut–brain communications. The microbiota-derived SCFAs can have an impact on host health as part of the metabolic signaling network (Renwick and Stewart, 2022). It has already been investigated that this microbial metabolite can influence immunoregulation and inflammatory modulation (Rooks and Garrett, 2016). SCFAs can regulate the innate immune cells, such as neutrophils, dendritic cells (DCs), and microglia, as well as the adaptive immune system, and can subsequently affect the inflammatory process (Erny et al., 2015; Corrêa-Oliveira et al., 2016). By promoting the production of cytokines, such as TNF-α, SCFAs can directly modulate the differentiation of neutrophils (Rodrigues et al., 2016). Considering the abovementioned inhibitory effect of SCFAs on HDAC, SCFAs can suppress the expression of the key transcription factors, which would consequently block the maturation of macrophages, monocytes, and DCs and weaken their ability to produce inflammatory cells or cytokines (Chang et al., 2014; Corrêa-Oliveira et al., 2016). SCFAs can also modulate the adaptive immune system; for example, they may influence the differentiation of regulatory T (Treg) cells (Tanoue et al., 2016; Dalile et al., 2019). Treg cells, marked by the expression of the transcription factor FOXP3, play a major role in suppressing unnecessary immune reaction and maintaining homeostasis (Veltkamp et al., 2006; Moes et al., 2010). The differentiation, maintenance, and migration of Treg cells are regulated by numerous signals that are mostly offered by the microbiota (Tanoue et al., 2016). SCFAs can activate Treg cell induction through histone H3 acetylation of the intronic enhancer named conserved non-coding sequence of Foxp3 by inhibiting HDAC activity (Arpaia et al., 2013; Furusawa et al., 2013). SCFAs can also directly promote the induction of Treg cells by G protein-coupled receptor 43 (GPR43) (Smith et al., 2013). Another mechanism for the induction of Treg cells is where butyric acid binds with GPR109A in DCs and promotes the expression of aldehyde dehydrogenase, which can induce the conversion of naive T cells into Treg cells (Singh et al., 2014). As SCFAs can migrate and follow the bloodstream, they may regulate the inflammation in the central nervous system based on these abovementioned mechanisms. In a neonatal hypoxic–ischemic rat model, sodium butyrate reportedly reduced the damage in the brain by inhibiting the production of IL-1β and the chemokine CXCL10, which could lead to hypomyelination and WMI (Jaworska et al., 2017). Sodium butyrate also reportedly shifted the microglia to the inflammatory inhibition phenotype (M2) in a central nervous system injury model through the HDAC effect (Jaworska et al., 2017, 2019). The above evidence supports that microbiota and their metabolites could potentially regulate the inflammation in the brain of very preterm babies, as shown in Figure 2.

## Potential Microbial Dependent Therapy to White Matter Injury in Very Preterm Babies

Probiotics are live microorganisms that can offer health benefits to the host (Martin and Walker, 2008). Probiotics may attenuate WMI in very preterm babies as they can shift the gut microbiota and, subsequently, regulate the immune system and inflammation response, which may also be correlated with attenuating WMI, as previously introduced (Bermudez-Brito et al., 2012). A recent report showed that administration of *Lactobacillus acidophilus* and *Bifidobacterium infantis* to pregnant mice could promote OL progenitor development, suppress IL-1β-induced systemic inflammation, and attenuate microglial activation in the offspring (Lu et al., 2020). Various clinic trials have been performed to explore the function of probiotics in very preterm babies. A reduction in the rate of onset of NEC was observed with the prophylactic use of probiotics in very preterm babies (Bin-Nun et al., 2005; Lin et al., 2008; AlFaleh and Anabrees, 2014). Since adverse neurodevelopment is associated with the onset of NEC, it is reasonable to speculate that probiotics may attenuate the neural injury (Shah et al., 2008; Adams-Chapman, 2018; Niño et al., 2018; Sun et al., 2018). Three randomized controlled clinical trials have been carried out to assess the effect of probiotic supplementation on the improvements of neurodevelopment. With a total of 1,210 patients enrolled (birth weight < 1,500 g or gestational age < 33 weeks), none of the studies reported a significant improvement in neurodevelopmental outcomes of the infant (Chou et al., 2010; Sari et al., 2012; Jacobs et al., 2017). However, in an observational study, the administration of probiotics for neonates was suggested to reduce rotavirus-associated WMI (Yeom et al., 2019).

Prebiotics are another kind of microbial nutritional components and can regulate the gut microbiota by favoring the growth of beneficial bacteria, such as *Bifidobacteria* (Davani-Davari et al., 2019). Prebiotics could regulate immune cells, including T cells, neutrophils, and DCs, to maintain immune balance and regulate inflammation response, which could subsequently influence the myelination and WMI in very preterm babies (Eiwegger et al., 2004; Eiwegger et al., 2010; Jeurink et al., 2013). Thus, prebiotics are presumed to attenuate WMI. To the best of our knowledge, two studies have explored the improvements in neurodevelopmental outcomes after prebiotic supplementation, and both have failed to show a significant benefit (LeCouffe et al., 2014; van den Berg et al., 2016). Due to the limited data published in this regard, it is difficult to judge whether probiotics or prebiotics can improve neurodevelopmental outcomes and, therefore, further studies are needed to clarify their function in neurodevelopment.

Thus far, the clinical trials of probiotics and prebiotics have failed to demonstrate an ability to reduce WMI in very preterm babies, and several factors may contribute to these unexpected results. First, probiotics were given late (on the seventh day after birth), and WMI may already occur by this time (Chou et al., 2010). Second, the low incidence of NEC and other predictors of WMI in these studies may obscure the effect of probiotics or prebiotics with small study populations (Chou et al., 2010; Sari et al., 2012; LeCouffe et al., 2014; van den Berg et al., 2016; Jacobs et al., 2017). Third, the administration of single-strain probiotics rather than multistrain probiotics may partly explain the lack of effect for probiotics (Sari et al., 2012). Due to these limitations of these published data, it is difficult to reach a verdict on whether probiotics or prebiotics can improve neurodevelopmental outcomes, and further studies are needed to clarify their function with a larger population and optimal administration time.

## Conclusion

The WMI is a multifactorial disease. Accumulating evidence has shown the role of microbiota in WMI; however, the present evidence is inadequate to establish a causative direct link between microbiome and WMI. Nevertheless, detrimental exposures during the prenatal or postnatal period, such as maternal infection, early nutritional intake, and medical interventions in NICU, which cause modifications to microbiome in very preterm infants, are all triggers of WMI. These modifications to microbiota also reflect the variation of extrinsic and intrinsic environments and are proposed to be the pivotal intermediate links. Since gut microbiota may play a critical role in the pathogenesis of WMI, modifications to microbiome in very preterm infants may be a promising target for WMI treatment. In addition, the potential role of the mother and her microbiome (not only vaginal but also oral mucocutaneous microbiome) should be emphasized here, due to the maternal role in infant microbial colonization, beginning with the transfer of microbes *in utero* and continuing its effect through breastfeeding in the early life. The gut–brain axis is regarded as a mediator of myelination, and apart from immunological pathway, it also includes endocrine and vagal pathways (Cryan and Dinan, 2012). For example, hypothalamic–pituitary axis, a classical neuroendocrine pathway, remains unexplored in the context of WMI. Reports have shown that maternal separation could influence the hypothalamic–pituitary–adrenal axis and myelination *via* the blockade of OL differentiation (De Palma et al., 2015; Yang et al., 2016). This study also showed that intestinal dysbiosis displayed an exaggerated response of the hypothalamic–pituitary axis (De Palma et al., 2015). The result corresponds to that of another study, wherein the normal microbiota were reported to regulate the response of hypothalamic–pituitary axis (Sudo et al., 2004). These limited data all indicate that more different avenues which may offer more comprehensive interpretations of the role of not only the gut immunological communication but other mechanisms should be explored in WMI in the future.

## Author Contributions

YH and YZ wrote the draft of the review. FL and YS have reviewed and revised the review and approved the final version before submission. All authors contributed to the article and approved the submitted version.

## Conflict of Interest

The authors declare that the research was conducted in the absence of any commercial or financial relationships that could be construed as a potential conflict of interest.

## Publisher’s Note

All claims expressed in this article are solely those of the authors and do not necessarily represent those of their affiliated organizations, or those of the publisher, the editors and the reviewers. Any product that may be evaluated in this article, or claim that may be made by its manufacturer, is not guaranteed or endorsed by the publisher.

## Abbreviations

WMI: white matter injury
Pre-OLs: pre-myelinating oligodendrocytes
OL(s): oligodendrocyte(s)
OPC(s): oligodendrocyte precursor cell(s)
PAMPs: pathogen-associated molecular patterns
DAMPs: damage-associated molecular patterns
NICU: neonatal intensive care unit
SCFAs: short-chain fatty acids
BBB: blood–brain barrier
MAMPs: microbially-associated molecular patterns
HDAC: histone deacetylases
IL-1 β: interleukin-1 β
TNF: tumor necrosis factor
PDGF-AA: platelet-derived growth factor-AA
VEGF: vascular endothelial growth factor
IGF-1: insulin like factor-1
DCs: dendritic cells
Treg: regulatory T cells
FOXP3: forkhead/winged-helix transcription factor3
GPR: G Protein-Coupled Receptors
CXCL10: chemokine (C-X-C motif) ligand 10
GF: germ-free
NEC: necrotizing enterocolitis.
